# Molecular epidemiology of HTLV-1 infection in the caribbean area as compared to West Africa : relationship with the slave trade

**DOI:** 10.1186/1742-4690-8-S1-A90

**Published:** 2011-06-06

**Authors:** Alexandra Desrames, Olivier Cassar, Philippe V Afonso, Olivier Gout, Olivier Hermine, Antoine Gessain

**Affiliations:** 1 Unité EPVO, Institut Pasteur, CNRS URA 3015, Paris, France; 2 Service de Neurologie, Fondation Rothschild, Paris, France; 3 Service d Hématologie, Hôpital Necker, Paris, France

## Introduction

HTLV-1 molecular epidemiological studies demonstrated its great genetic stability, with different subtypes/subgroups, linked to the geographical origin. These peculiar features can be used as molecular means to study migrations of HTLV-1 endemic populations. Although the Caribbean archipelago is a high HTLV-1 endemic area, little is known concerning the HTLV-1 genetic diversity in this region.

## Materials and methods

HTLV-1 infected persons, either patients or HTLV-1 asymptomatic carriers, mostly living in Paris area, were included. Their geographical origin was determined through interviews. DNAs, extracted mostly from PBMCs were subjected to PCRs amplifying two LTR fragments. PCR products were sequenced and phylogenetically analyzed.

## Results

112 persons were included with 55 from West-Indies, 26 from French Guyana, 23 from West Africa and 8 from Central Africa. Comparison of the complete LTR indicated that most of the West African strains were of the Senegal subgroup or West African one (AWA). In contrast and surprisingly, all West Indian strains, except one, belong to the Transcontinental (TC) subgroup of the Cosmopolitan subtype. Interestingly, half of the French Guyanese were infected by a TC strain while the others (all of Noir Marron origin) were infected by an AWA strain.

## Conclusion

Further studies combining a larger number of samples from West Indian and West Africa, together with precise historical data are ongoing to gain better insights into the origin and modes of dissemination of HTLV-1 in the Caribbean and surrounding areas, as well as the origin of these populations in Africa.

